# A Flexible PDMS-Based Optical Biosensor for Stretch Monitoring in Cardiac Tissue Samples

**DOI:** 10.3390/s23239454

**Published:** 2023-11-28

**Authors:** Andrea Sannino, Antonio Velarte, Aránzazu Otín, José Ignacio Artigas, Aida Oliván-Viguera

**Affiliations:** 1 Group of Power Electronics and Microelectronics, Aragon Institute for Engineering Research, University of Zaragoza, 50018 Zaragoza, Spain; aranotin@unizar.es (A.O.); jiartigas@unizar.es (J.I.A.); 2 Biomedical Signal Interpretation and Computational Simulation Group, Aragon Institute for Engineering Research, Aragon Health Research Institute, University of Zaragoza, 50018 Zaragoza, Spain; avelarte@unizar.es; 3 CIBER-BBN, Biomedical Signal Interpretation and Computational Simulation Group, Aragon Institute for Engineering Research, Aragon Health Research Institute, University of Zaragoza, 50018 Zaragoza, Spain; aolivanv@unizar.es

**Keywords:** PDMS, strain sensor, biosensor, optical fiber, cardiac applications, biomimetic culture chamber, organ-on-chip

## Abstract

Cardiotoxicity, characterized by adverse impacts on normal heart function due to drug exposure, is a significant concern due to the potentially serious side effects associated with various pharmaceuticals. It is essential to detect the cardiotoxicity of a drug as early as possible in the testing phase of a medical composite. Therefore, there is a pressing need for more reliable in vitro models that accurately mimic the in vivo conditions of cardiac biopsies. In a functional beating heart, cardiac muscle cells are under the effect of static and cyclic stretches. It has been demonstrated that cultured cardiac biopsies can benefit from external mechanical loads that resemble the in vivo condition, increasing the probability of cardiotoxicity detection in the early testing stages. In this work, a biosensor is designed and fabricated to allow for stretch monitoring in biopsies and tissue cultures using an innovative sensing mechanism. The detection setup is based on a biocompatible, thin, flexible membrane—where the samples are attached—which is used as an optical waveguide to detect pressure-caused shape changes and stretches. Various prototypes have been fabricated with a cost-effective process, and different measurements have been carried out to experimentally validate the proposed measurement technique. From these evaluations, stretches of up to 1.5% have been measured, but the performed simulations point towards the possibility of expanding the considered technique up to 10–30% stretches.

## 1. Introduction

Polymers and flexible substrates are considered to be of key importance in many fields and applications. For instance, flexible materials are largely employed in the design and implementation of biosensors thanks to their malleability and elasticity [1,2,3,4]. These properties allow for polymer-based sensors to have a wide range of applications that include tissue/cell culturing [5], e-skin [3,6,7,8], wearable electronics and devices [9,10], self-healing robots [11], Human–Machine Interfaces [12,13,14], and industral applications [15,16]. In all these applications, the design is based on a soft and flexible substrate able to measure stretch under the appropriate load conditions. The most-used materials are polydimethylsiloxane (PDMS), polymethylmethacrylate (PMMA), and graphene.

Technology-wise, stretches and deformations are typically measured with strain gauges [17], where the pressure applied to the material is translated into a change in electrical resistance [10]. Even though this technique has proven to be as a very effective solution for in situ stretch monitoring, the photolithography or deposition of other materials like graphene is needed, adding complexity to the fabrication process.

Capacitive strain sensors are another widely spread class of devices used for the real-time monitoring of deformations. In this technology, an insulating layer is manufactured between two conductive electrodes. Applying a certain strain to the electrodes, the capacitance is modified due to the change in the geometrical area of the equivalent capacitor, enabling the readout of strain [18]. Furthermore, piezoelectric and triboelectric materials are also printed on flexible substrates for strain-sensing, obtaining high precision and fast stretch measurements [19,20,21].

Another very important class of sensors is the Fiber Bragg Grating (FBG)-based sensor. In these implementations, the sensing device consists of an optical fiber whose refractive index has been externally modified, or one in which some impurities have been added to the fiber structure [22]. By analyzing the output radiation, it is possible to quantify the stretch/strain that is applied to the fiber. The majority of the aforementioned techniques, independently of the application, involve the use of a polymeric composite as a structural material. More specifically, a thin polymeric layer is often used as a matrix for the fabrication of the necessary sensing elements.

Some proposals based on these ideas have recently been developed, considering PMMA as a structural material. The sensor proposed in [23] was based on the mechanical movement of two optical fibers that were adjacent under rest conditions. When subjected to an external stretch, they became distanced, resulting in a loss of light intensity due to the alterations in the refractive index. In this work, an innovative sensing principle is presented where the polymeric thin layer is used as a sensing layer, employing it as an optical waveguide and using the principle of Total Internal Reflection (TIR) to detect stretches. After defining the operating principle, the mechanical structure is mathematically modelled and then validated through software simulations. Afterwards, the device is specialized for cardiac tissue and cellular growth and maturation applications, adapting its functionalities to the field requirements. Finally, a thick-film, cost-effective fabrication method is presented and measurements are carried out to validate the new sensing principle and test its suitability for the aforementioned application.

## 2. Stretch Sensor: Design and Fabrication

In this section, an innovative sensing principle is proposed and the sensor’s mechanical structure is designed. Mathematical models are used to predict the geometry’s behaviour and simulations are carried out to validate the design. Finally, the thick-film, low-cost fabrication process is presented.

### 2.1. Stretch Application and Sensing Mechanism

**Figure 1:**
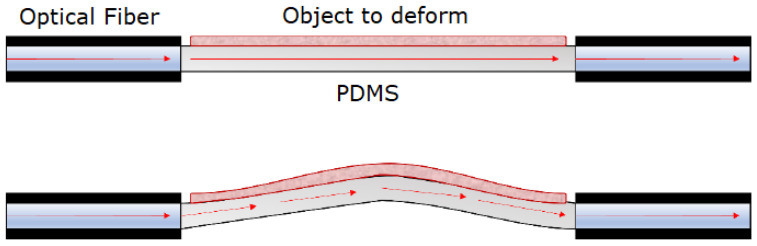
Sensor working principle. Radiation (red arrows) is guided through the thin membrane that deforms; recollected light carries information about the deformation.

To measure stretch, a mechanism was required to induce deformation on a flexible thin membrane, where the object of interest was situated. In this particular instance, pressure was applied from below, resulting in an upwards bending of the flexible substrate, which caused a longitudinal elongation of the fixed element, as visible in Figure 1. The consequential membrane shape change could be employed for detecting the induced elongation through optical measurements, using the membrane as an optical waveguide. Specifically, when no pressure was exerted, the TIR condition was met, resulting in maximum optical transmittance. On the other hand, when pressure deformed the flexible layer, the TIR condition was gradually lost and the membrane optical transmittance lowered accordingly, resulting in a lower optical intensity being received at the other membrane end.

PDMS was chosen as structural material for the sensor due to its biocompatibility, flexibility, low-cost manufacturing and transparency in visible light. For the optical measurements, two commercial plastic optical fibers (POF) (MH4001 EskaTM, 1.0 mm Core Simplex High-Performance Plastic Optical Fiber, 2.2 mm OD Polyethylene Jacketed, Industrial Fiber Optics, Tempe, AZ, USA) were positioned at the sensor’s lateral faces in correspondence with the active layer. Hence, the decision was made to set the thickness of the active membrane at 1 mm, ensuring a consistent optical path.

### 2.2. Sensor Structural Design

**Figure 2:**
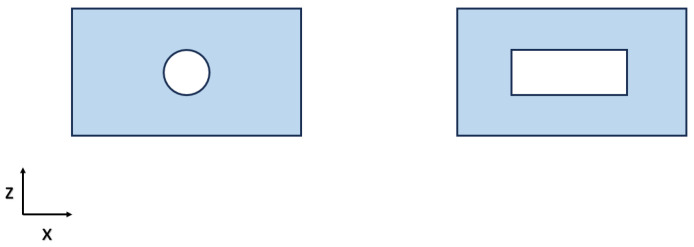
Cross section of two possible sensor geometries.

**Figure 3:**
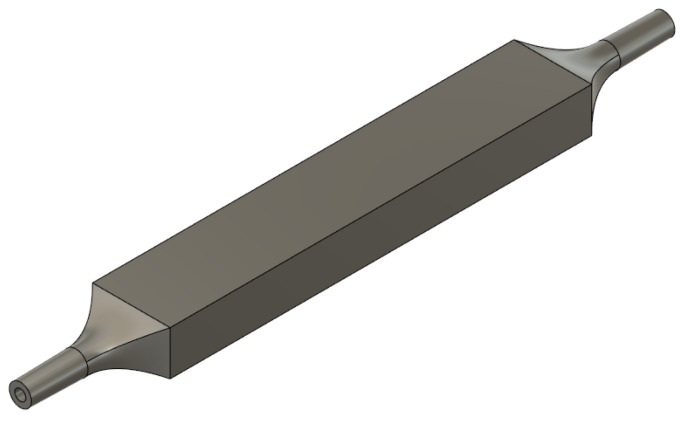
3D model of sensor with the two PLA funnels attached to it.

A hollow rectangular PDMS structure was designed to mechanically support the active membrane and allow for the application of pressure. Two different possible geometries were investigated, as shown in Figure 2. In both designs, a cavity was embedded for air injection. The shape of the cavity and the thickness of the upper layer are pivotal structural parameters, as they dictate the pressure that is required to achieve the intended stretch. Moreover, the upper layer thickness has to be paired with the optical fiber diameter to allow for impedance-matching between the two waveguides, reducing power losses at the interface. Thus, the thin sensing layer was designed to be of 1 mm thickness as the optical fiber diameter. Following a thorough analysis, the prototype featuring a circular cavity was rejected due to the pressure’s inability to cause a significant displacement of the upper layer, rendering this prototype ineffective. The geometry was selected to be of dimensions 76 × 14 × 8 mm so that the air boundary conditions at the inlets and at the central part would be separated, achieving a more uniform deformation of the membrane part that adheres with the object of interest. To facilitate pressure application, two identical Polylactic Acid (PLA) funnels were designed using Fusion 360 (Autodesk, Mill Valley, CA, USA), 3D-printed, and glued to the structure. Figure 3 shows a 3D model realized with Fusion 360 of the whole structure with embedded funnels.

### 2.3. Mechanical Design Model and Validation

**Figure 4:**
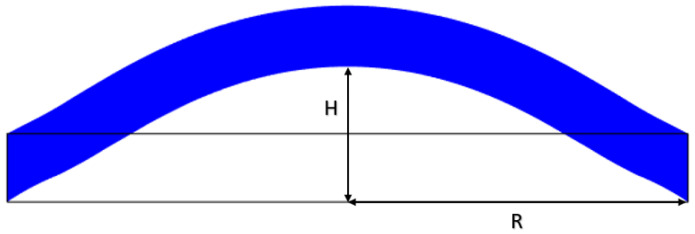
Schema of the vertical displacement for strain calculations.

The main goal of this section is to study the mechanical properties of the 1 mm membrane and to demonstrate that the stretch values can occur within the range of interest. A mathematical model was used to predict the required vertical displacement and strain on the membrane that is achievable with this system architecture. Figure 4 represents a schema of the problem. $L_{0}+\Delta L$ is the longitudinal increased length of the sensitive membrane, H is the vertical displacement under the pressure effect and R is half of the cavity width—in this case, 5 mm.

Using the schema shown in Figure 4, the strain ϵ can be calculated as in [24]:

(1) $$\epsilon =\frac{\Delta L}{L_{0}}=\frac{R^{2}+H^{2}}{2RH}arcsin\left(\frac{2RH}{R^{2}+H^{2}}\right)-1.$$

**Figure 5:**
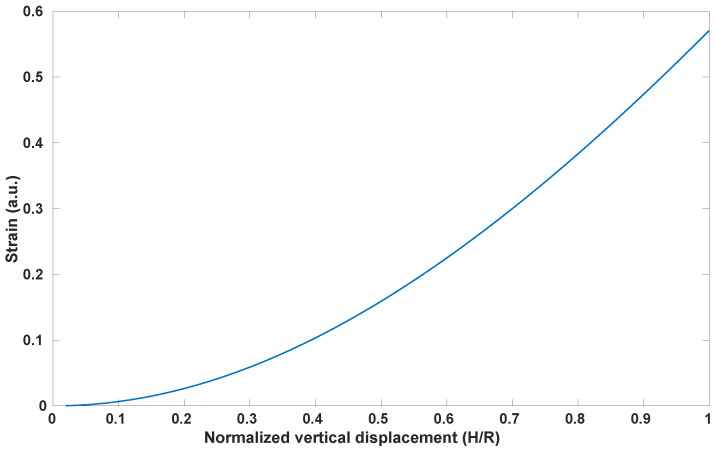
Strain vs. Normalized Vertical Displacement (Equation (1)).

In a first instance, Equation (1) was used to graph the strain with the normalized vertical displacement H/R. From Figure 5, it is possible to notice that, to achieve a strain—and thus a stretch—of 10–30%, a vertical displacement of H = 2–3.5 mm is needed.

**Figure 6:**
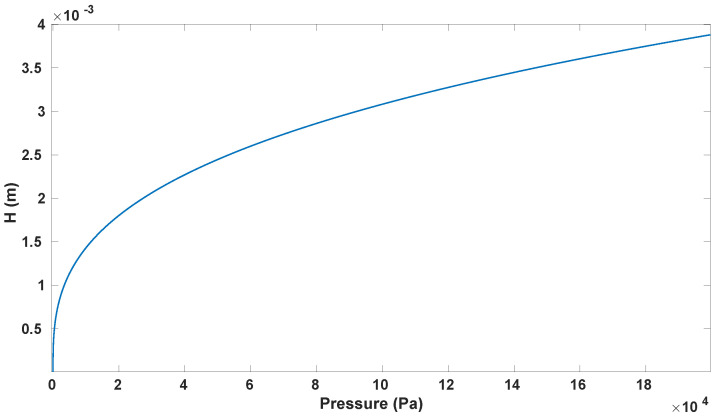
H vs. pressure according to the Bulge Test equation (Equation (2)) for the device membrane.

The Bulge Test Equation [24] was utilized to determine the pressures at which these displacements occur:

(2) $$P=\frac{2\sigma_{0}t}{a^{2}}H+\frac{4Et}{3a^{4}\left(1-\nu^{2}\right)}H^{3},$$

where 2a is the membrane width, $\sigma_{0}$ is the initial stress, *t* is the membrane thickness, and *E* and ν are the Young’s modulus and the Poisson’s ratio of the membrane, respectively. Since, in this case, the initial stress was null, the equation was reduced to:

(3) $$P=\frac{4Et}{3a^{4}\left(1-\nu^{2}\right)}H^{3},$$

from this, the displacement can be retrieved:

(4) $$H=\sqrt[{3}]{\frac{3a^{4}\left(1-\nu^{2}\right)P}{4Et}},$$

where, for the proposed structure, a=5 mm, t=1 mm, ν=0.5 and the Young’s Modulus of PDMS (*E*) is 1.2 MPa [25]. From Figure 6, it is possible to infer that, to obtain a vertical displacement H=2 mm (10% stretch), a P=27 kPa is needed. However, to obtain H=3.5 mm, corresponding to a stretch of 30%, a P=150 kPa is needed.

To corroborate these statements, a COMSOL Multiphysics (COMSOL Inc., Burlington, MA, USA) simulation was carried out. To simulate the scenario, only the 1 mm active layer was considered in the model and a constant pressure was applied to the lower membrane surface. After drawing the geometry, selecting the material, and adding the appropriate equations, a parametric sweep of the applied pressure was performed.

**Figure 7:**
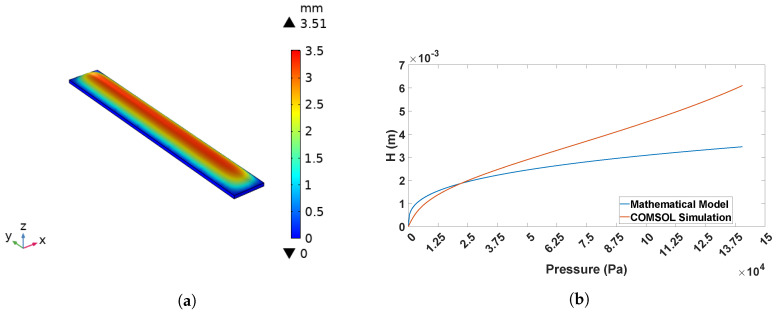
COMSOL Simulations of the membrane cyclic loading test. (**a**) 3D results of the vertical displacement COMSOL simulation. (**b**) Comparison between the Bulge Test Equation and the hyperelastic model simulated with COMSOL.

Figure 7a shows the 3D result of this parametric sweep. In order to increase the simulation accuracy, the hyperelastic properties of PDMS were added to the COMSOL material model. The results are presented in Figure 7b, where it is possible to notice that stretches in the 10–30% range could be achieved with pressures in the range 25–70 kPa.

### 2.4. Stretch Monitoring Applied to Cardiac Research

In the field of pharmaceuticals, new drug compounds undergo multiple stages of testing throughout their development process prior to being introduced to the market. After the discovery of a new compound, various preclinical and clinical testing phases are required. One of the requirements is the evaluation of cardiotoxicity of a drug, which can alter the normal functioning of the cardiomyocytes (human cardiac cells) and cause life-threatening arrhythmia. During the pre-clinical phase, tests are carried out using models that closely resemble the human cardiac condition (in vivo), excluding human participants from the experiments. Preclinical models involve:

- Animal models.
- In silico models: software simulations of the cardiac structure under the compound effect.
- In vitro models: 3D tissue slices or human-induced pluripotent stem-cell-derived cardiomyocytes (hiPSC-DM) that are cultured and kept under standard conditions.

When extracted from the functional organ, cardiomyocytes maintain their functional structure and behaviour. However, some time after isolation, these cells start to change their structural properties and functionality [26,27]. To stop this degrading, it is of key importance that the slice is cultured under conditions that mimic the in vivo situation. These conditions are:

- Continuous electrical stimulation.
- Mechanical loading.
- Oxygen and nutrients supply.

Electromechanical stimulation has to be applied with external systems, while the oxygen and nutrient supply is guaranteed by placing the slice in contact with an appropriate salt solution or culture medium that mimics native extracellular fluid (ECF) [28]. For this reason, Biomimetic Culture Chambers (BMCCs)—where all these physiological conditions are met—are needed for the appropriate culturing of both cardiomyocytes and tissue slices. In various studies [29,30,31,32,33], BMCCs have been developed to investigate the effect of mechanical stimulation protocols over cardiomyocytes and cultured slices. The main findings were that the best static mechanical stretch was 20–30% of the original length, corresponding to a sarcomere length (SL) of 2.2–2.4 μm. With these mechanical loads, tissue slices were successfully cultured and kept alive for up to 4 months. In [5], stretch measurements of up to 4–5% were obtained using a microfabricated device with strain gauges, and cardiomyocytes were successfully cultured under such stretch conditions. However, in [30], stretch measurements were carried out employing a digital camera and a millimeter graph paper; thus they lacked resolution, requiring expensive equipment and extensive calibration and data post-processing. The device proposed in [5] needs fabrication that can produce such thin-film substrates and the equipment required to read the sensor output. For these reasons, advanced measuring techniques are required to enhance the design of BMCC and optimize stretch monitoring effectiveness, and a cost-effective, widely available fabrication process has to be introduced for such devices. The presented measurement method is proposed as an affordable alternative to the state-of-the-art sensing mechanisms, and tests on various prototypes with different properties are carried out to verify its suitability. The cardiac biopsies taken into account for this study were of the dimensions 5 × 7 × 0.3 mm.

### 2.5. Mold and Sensor Fabrication

**Figure 8:**
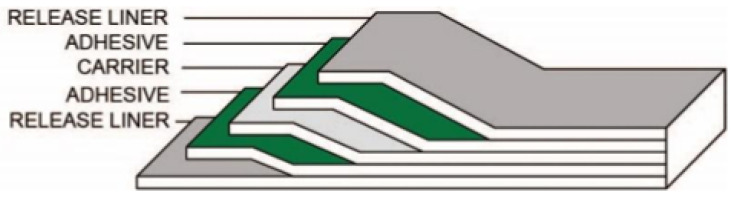
PSA ARcare^®^ 8939 structure [34].

As a first fabrication step, the molds were designed and fabricated according to the fixed structural dimensions. The chosen mold material was PMMA. After creating a Corel Draw v.20 (CAD) 3D model, the mold was manufactured using a ${\mathrm{CO}}_{2}$ laser printer (Epilog Mini 24, Epilog Laser, Golden, CO, USA). PMMA foils were adhered with a pressure-sensitive adhesive (PSA) ARcare-8939 (Adhesive Research Inc., Glen Rock, PA, USA). Figure 8 shows the PSA structure. PSA and PMMA were chosen for their robustness, low-cost manufacturing and suitability for clinic devices and applications. Furthermore, in order to realize the internal sensor cavity, the same PMMA/PSA process was used to fabricate a rectangular bar.

Afterwards, to facilitate demolding, a silane pre-treatment of 2 h was performed on the mold. A total of 10 μL of silane ${\mathrm{CF}}_{3}{\left({\mathrm{CF}}_{2}\right)}_{5}{\left({\mathrm{CH}}_{2}\right)}_{2}{\mathrm{SiCl}}_{3}$, 97% (ThermoFisher GmbH, Kandel, Germany) was applied close to the mold under vacuum conditions in order to form a uniform thin cap on the container surface.

The third manufacturing step was to actually realize the polymer. For this purpose, Sylgard-184 silicone elastometer (The Dow Chemical Company, Midland, MI, USA) was put into use. The base and curing agent were put into a standard laboratory mixer with a 10:1 ratio and manually mixed until homogeneity was achieved. Three different fabrication steps are possible:

1. Evacuation of the silicone encapsulant in a vacuum chamber for 3 h to remove bubbles and subsequent drying heat treatment at 70 ${}^{\circ }$C for 1 h.
2. Centrifugation at 1200 rpm for 5 min of the container and the encapsulant to eliminate entrapped air and 3 h of 70 ${}^{\circ }$C heat-curing.
3. Mold with encapsulant left at room temperature for 48 h.

**Figure 9:**
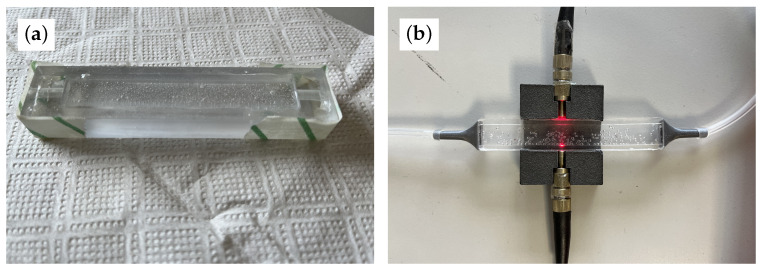
Representation of the molding process and completed and mounted sensor. (**a**) PDMS in mold before heat processing and top layer bubble removal. (**b**) PDMS sensor after fabrication with light and pneumatic circuit.

All three proved successful at removing bubbles from the sensing membrane (i.e., the 1 mm layer where light passes); however, the second produced more consistent sensors in terms of density, texture, and opacity, so the other two methods were discarded. Figure 9a shows the mold with the polymeric solution, while Figure 9b shows how light is injected from the optical fiber to the PDMS sensor. Three different prototypes were fabricated, with different structural properties, to validate the sensing system, as follows:

- Prototype 1: Sensing layer of 1 mm and funnels glued with Loctite Super Glue-3^®^.
- Prototype 2: Sensing layer of 1 mm and funnels glued with Pattex Repair Extreme^®^.
- Prototype 3: Sensing layer of 1.2 mm and funnels glued with Pattex Repair Extreme^®^.

Pattex Repair Extreme^®^ was explored as an alternative glue since it provided a better bonding between the funnels and the PDMS sensor thanks to its polymer-like bond-creation properties. Loctite Super Glue-3^®^ was proved to create a less reliable contact that was often subject to pressure losses and detachment, invalidating the respective prototypes. For this reason, and as seen in the characterization results, prototype 1 was not further taken into consideration.

## 3. Measurements Setup

The measurement setup is presented in this section. In the first instance, optical path characterization was performed to understand the optical behaviour of the membrane. Once its suitability as an optical waveguide was determined, and the wavelengths for which the transmittance is at its maximum were found, the best optical components for the application were chosen. Afterwards, the mechanical setup to accommodate the sensor and align the optical fibers was studied and fabricated, taking into account the typical tissue and BMCC dimensions. Furthermore, the hardware and software setup is presented and the electronic measurement techniques are discussed.

### 3.1. Optical Path Characterization

To choose the best radiation parameters for the application, an optical path characterization was carried out. This experiment consists of emitting a broadband light radiation into the 1 mm active sensing layer and recording the received light intensity. In this way, one could understand which wavelengths cause the material to be transparent. These wavelengths will be =used to select the most appropriate optoelectronic components for the proposed system. The broadband light source used for this experiment was HL-2000 (Ocean Insights, Orlando, FL, USA) and the spectrometer was QE6500 (Ocean Insights, Orlando, FL, USA).

**Figure 10:**
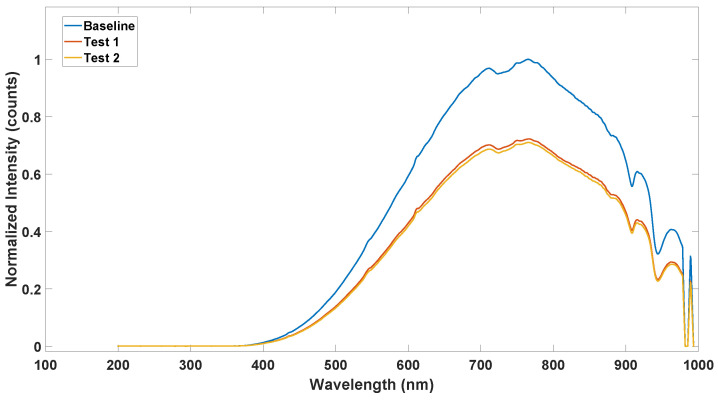
Optical path characterization. Baseline represents the results obtained for 0 Pa pressure, Test 1 and Test 2 for 100 and 200 Pa, respectively, to show that only the recollected light intensity changed when applying strain.

Figure 10 presents the results of this spectroscopy, realized in the UV/Visible (λ = 200–1000 nm) band.

The *Baseline* graph shows the results of the test when no pressure was applied and the membrane was in its resting position. Test 1 and Test 2 show the results of the same experiment but when pressure was applied to the sensing layer, causing its shape to change. As expected, the membrane bending caused a loss of the TIR condition, and less light intensity was recollected, but no variation was recorded in the frequency band. The blue baseline graph is the average of multiple measurements performed to test the repeatability, and a maximum normalized standard deviation of SD = 0.01 was recorded. Seeing these results, an FB00AKAR (Firecomms, Lehenagh More, Ireland) optocoupler was selected for the emission and recollection of radiation in the prototype. This optocoupler features a red LED that emits within the wavelength range of interest and has a high bandwidth, low capacitance, and separated photodiode.

### 3.2. Mechanical Setup

The mechanical setup consisted of mainly two parts:

1. Pneumatic circuit.
2. Sensor mechanical support.

**Figure 11:**
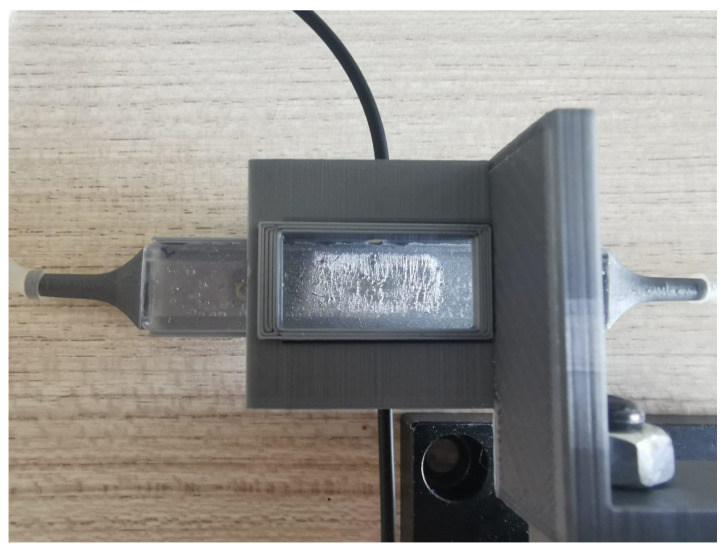
Final mechanical setup. The two fibers are always aligned and the support mimics the typical shape of a biomimetic culture chamber. Height was adjusted to improve alignment with a micrometer screw.

The pneumatic circuit was formed by a syringe for controlled air injection and a series of silicone tubes, of 4 mm outer diameter and 2 mm inner diameter, that were connected to the sensor through its PLA funnels. In order to allow for proper fiber alignment to the 1 mm active layer, a platform incorporating a micrometer screw and the fiber accommodation holes was designed and 3D-printed. Moreover, a small chamber was placed on top to ensure that only the central membrane region deformed and to serve as a BMCC. With this setup, visible in Figure 11, it was possible to adjust the alignment of the optical fibers to the desired location, allowing for more repetitive measurements. To ensure proper alignment with the sensing layer, the micrometer screw was initially placed in a position where the radiation would not pass through the PDMS device. Thereafter, the fiber support was lowered until the first received light intensity maximum was recorded, guaranteeing that the light was guided by the device sensing layer. In this way, a repetitive measurement method was guaranteed for each prototype.

### 3.3. Electronic Signal Conditioning and Data Acquisition

**Figure 12:**
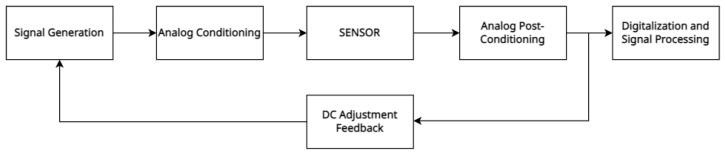
Electronic system architecture. The feedback loop maintains a constant DC level, desensitizing the measurements to ambient light variations.

**Figure 13:**
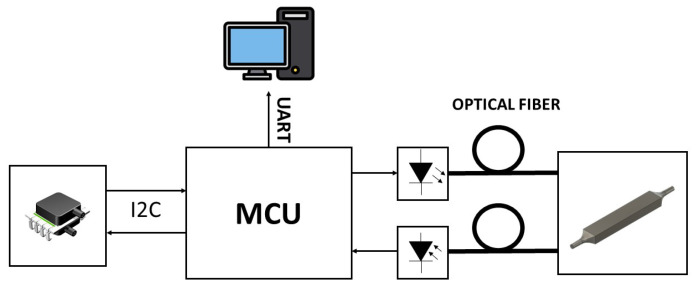
Characterization setup and communications. The MCU was communicating with an I2C protocol with a pressure sensor and with an UART protocol to send data to the PC.

Figure 12 and Figure 13 show a schema of the hardware/software architecture for optical sensing. The signal generation block was formed by a DAC, where a sine wave of 500 Hz was generated.

An appropriate amplification and further analog signal conditioning allowed for the appropriate LED polarization, generating the desired optical radiation. This optical signal was guided through the 1 mm thick sensing layer and collected with the photodiode. Both the LED and the photodiode were operating in their linear range. After adequate filtering and amplification, the signal carrying the information was digitized and post-processed to obtain low noise results. The ADC used for the data digitization was the one embedded in the microcontroller unit (MCU)—with 12-bit resolution—and samples were taken with $f_{s}=25$ kHz. Since the photodiode proved to be very sensitive to ambient light variations, the optical components had to operate under conditions that were robust to the DC variations that affected the measurements. Therefore, a feedback loop was introduced to implement a DC-level control over the excitation signal. In this way, the information regarding the membrane shape change was only encoded in the AC amplitude of the recollected signal at the photodiode, desensitizing the measurements from ambient light variations and other DC phenomena. The signal generation and digitization were fully developed with a Nucleo-L452RE-P board (ST Microelectronics, Geneva, Switzerland). A pressure sensor DLHRL10G (Allsensors, Morgan Hill, CA, USA) was employed for real-time pressure measurements.

Communications between the MCU, pressure sensor and PC were implemented with different communication protocols. The pressure sensor sent raw data to the MCU with the I2C communication protocol. Furthermore, the information recollected from the photodiode was digitized using the embedded ADC of the MCU. With UART protocol, strings containing the raw pressure data and intensity data were sent to the PC for further processing. Matlab (The MathWorks, Apple Hill Drive Natick, MA, USA) scripts were developed to synchronize the operations between the pressure sensor and the intensity data recollection system. These scripts were also employed to recollect, organize, and post-process the UART data.

## 4. Results

### 4.1. Voltage vs. Pressure Characterization

**Figure 14:**
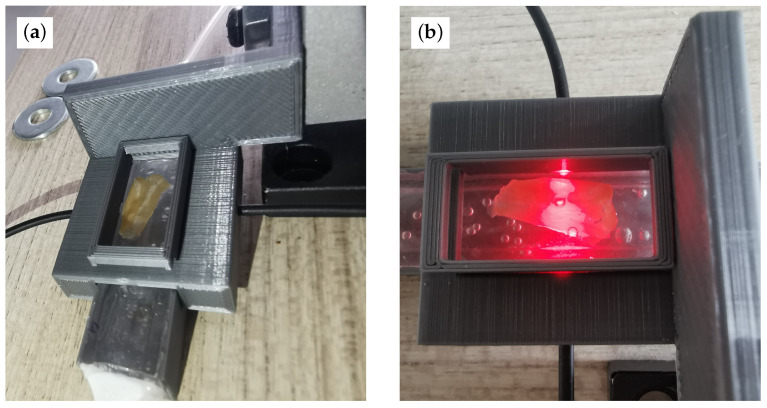
Measurement setup with heart sample allocated in the BMCC. To apply strain to the sample and guarantee its adherence to the top layer, the slice was glued to the PDMS substrate. (**a**) Inflated membrane. It is possible to observe how the cardiac sample is stretched. (**b**) Relaxed membrane with red light passing through the sensor.

The first characterization step was to find the relationship between the injected air pressure and an electrical quantity that could carry the information of the membrane deformation. The root mean square (RMS) voltage of the sine wave collected by the photodiode was selected. The AC amplitude of this signal varied based on the deformation of the sensing layer. Therefore, the RMS of this sine wave is a noiseless measure of the deformation amplitude. Figure 14 shows the setup configuration with an allocated cardiac tissue sample. Figure 14a shows how the stretch is induced in the cardiac fiber, and Figure 14b shows how light passes through the sensing membrane.

**Figure 15:**
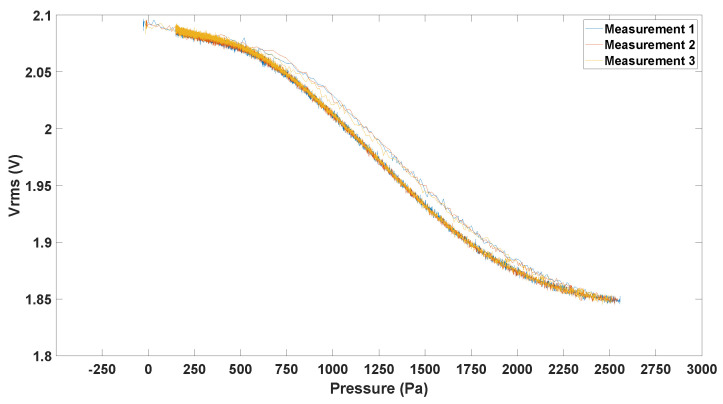
Vrms vs. pressure for prototype 3 during inflation and deflation.

The recollected signal from the photodiode was conditioned and digitized with the Nucleo-L452RE-P board. Raw data from the pressure sensor were sent to the MCU with the I2C protocol. Through a software interruption, data from the sine wave were digitized and, together with pressure data, were sent with the UART protocol to a PC. Once the data were sent to the PC, the dataset was saved in a text file and post-processed. In Figure 15, the relation between RMS voltage (Vrms) and pressure for prototype 3 can be seen. This measurement was first taken by inflating the membrane with the syringe and then letting it gradually deflate. As is evident, when observing the same pressure point, the Vrms measured during inflation and deflation differed, particularly within the high-sensitivity central region of this characteristic.

This observation indicates that the material exhibits an hysteresis that can be justified using the viscoelastic behaviour of PDMS [35]: due to creep, a viscoelastic material delays the complete recovery of its initial shape after a load is released. Nevertheless, due to the slower deflation rate and the availability of more measuring points, only this segment was taken into account when constructing the following Vrms–pressure relationships.

**Figure 16:**
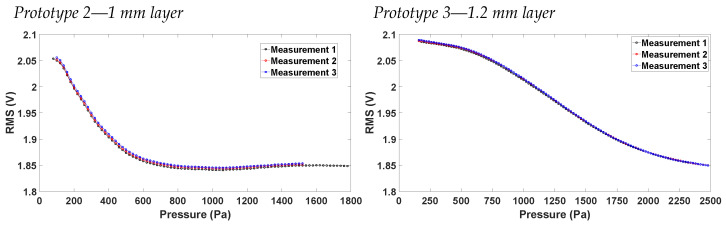
Vrms vs. pressure characterization for the two valid prototypes.

Figure 16 shows the characteristics of prototypes 2 and 3, respectively. As expected, the two curves differ in pressure range. This behaviour can be explained by the differences in the sensing layer thickness. In the case of prototype 3, light remains confined within the sensing layer for an extended duration during both inflation and deflation, resulting in a broader pressure range being reliably captured. However, it is anticipated that a higher pressure will be necessary for prototype 3 to achieve an equivalent vertical displacement compared to prototype 2.

### 4.2. Vertical Displacement Characterization

**Figure 17:**
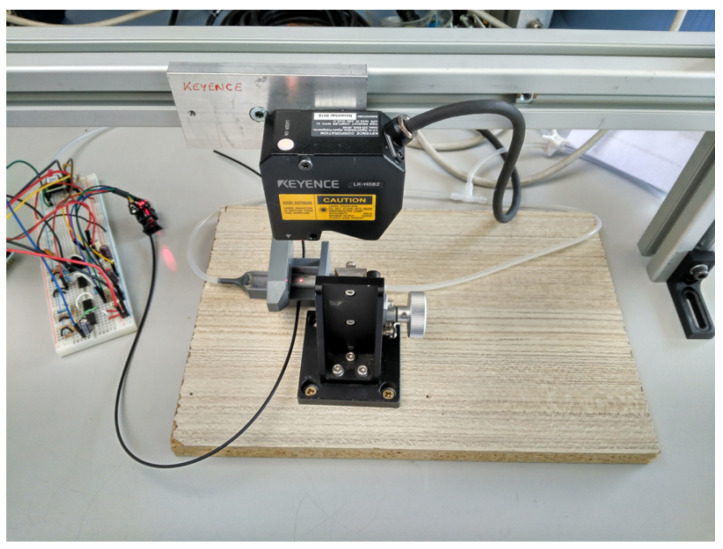
Measurement setup for vertical displacement characterization.

Having characterized the relation between Vrms and pressure, the vertical displacement of the membrane was measured. Using a vertical displacement laser LK-H082 (Keyence Corporation Of America, Itasca, IL, USA), and with the setup shown in Figure 17, the vertical displacement was related to the air pressure in the cavity. Since the PDMS used to produce the sensors was transparent to the laser wavelengths, the upper layer of the samples was painted with a grey paint to allow for proper reflections needed by the vertical displacement laser.

The optical fiber was disconnected from this setup because, otherwise, the direct characterization of Vrms vs. height would not be possible for two reasons:

1. The paint changes the way light is guided in the sensing layer. It appeared that light was better confined in the membrane, changing the Vrms vs. pressure behaviour.
2. Measuring the vertical displacement without painting the top layer was not possible, since PDMS is highly transparent under red light, preventing the proper vertical measurements and changing the Vrms characteristics due to interference from the laser light and fiberoptic radiation.

Following these arguments, only the circuit part, responsible for pressure measurements, was kept connected. Once the vertical displacement tool was properly focused and positioned at the membrane center, using a syringe, air was blown inside the cavity, causing the upper membrane to displace vertically.

Pressure points were taken within a sampling period of 60 ms, while the vertical displacement tool was set to capture height points with a sampling period of 20 ms. Thus, to relate pressure and displacement, a resampling process was necessary. Moreover, to better adjust these points, one of the two curves was shifted and compared to the other until the best correlation condition was met. Once this processing was finished, data cleaning and interpolation were performed to obtain height values at specific pressure points to allow for further characterization.

**Figure 18:**
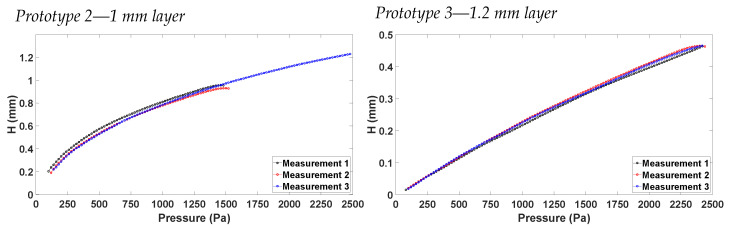
H vs. pressure characterization for the two valid prototypes.

In Figure 18, the vertical displacement H vs. pressure characteristics are shown for prototype 2 and prototype 3. Comparing the two prototypes for the same pressure points, prototype 3 always measured a smaller vertical displacement. This was expected, since the sensing layer of prototype 3 was fabricated to be thicker than that of prototype 2. Hence, more pressure was needed to achieve the same vertical displacement. Measurements were performed for up to 2.5 kPa, since this was the maximum pressure range of the commercial pressure sensor employed in the measurement.

### 4.3. Stretch Calculation

**Figure 19:**
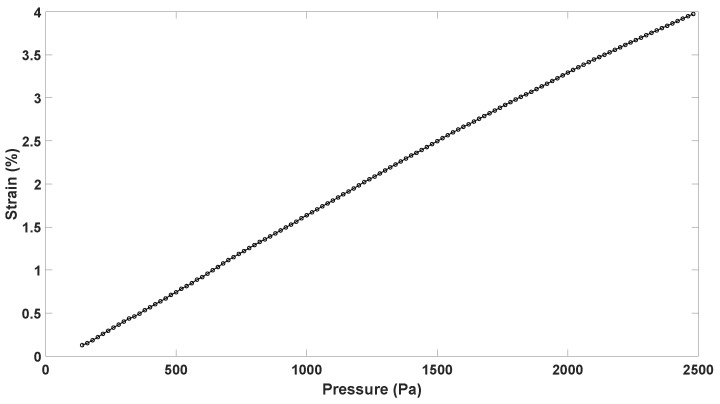
Strain vs. pressure curve for prototype 2.

Based on the sensor’s geometry and as the maximum vertical displacement that was achieved was for prototype 2, the strain-pressure characteristic was evaluated, as in Equation (1), although only for this prototype. From Figure 19, it is possible to see how, for a pressure of 2.5 kPa, a strain of 4% could be achieved, thus resulting in a 4% tissue stretch.

This results demonstrate that the structure can be used for cyclic mechanical stimulation for the maturation of cardiac microtissues since, in these stretch ranges, they remained functional for up to 4 days, as stated in [31].

### 4.4. Vrms vs. Displacement Characteristics

**Figure 20:**
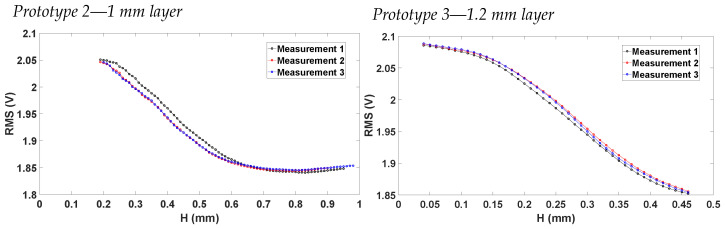
RMS vs. H characterization for the two valid prototypes.

Having characterized the Vrms and the vertical displacement with pressure, the relation between Vrms and height was found using various Matlab scripts. First of all, an interpolation of pressure points was carried out in a selected vertical displacement rang. Afterwards, the Vrms points were interpolated in these new pressure points. These interpolations and data processing allowed for the Vrms–height characteristic to be established. Figure 20 shows the results of this processing, where it is possible to notice that the 20% sensing layer thickness increase between prototype 2 and 3 influences the RMS-H characteristic, as expected. Moreover, the sensing technique proves to be able to measure stretches up to 1.5%.

**Figure 21:**
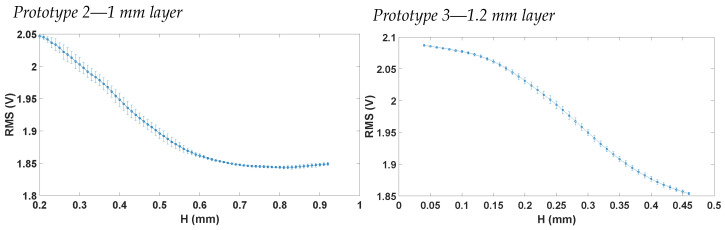
Mean and standard deviation for Vrms vs. H curves.

To test the repeatability of the two prototypes, the mean and standard deviation of the Vrms–height curves were calculated using a Matlab script from several measurements. These results are shown in Figure 21. For prototype 2, the maximum standard deviation was reported to be $\sigma_{max}=1.13\%$, while for prototype 3 $\sigma_{max}=0.61\%$, showing the very high repeatability of the two prototypes.

### 4.5. Impact of Painting on the Vrms Characteristic

As previously stated, in order to measure the membrane vertical displacement, the top layer needed painting to facilitate the laser reflection and allow for correct measurement. The painting does not affect the membrane displacement, as it is weightless compared to the membrane. However, paint was proven to change the Vrms–pressure characteristic.

**Figure 22:**
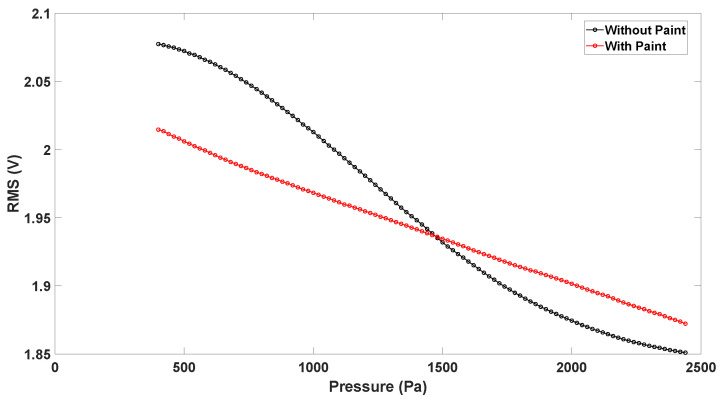
Variation in the Vrms–pressure curve when paint is applied to the device top layer.

In Figure 22, it is possible to notice how painting the membrane changes its optical behaviour.

The curve appears to be linearized by the painting, and the high-sensitivity region indicates a better confinement of the LED radiation to the 1 mm sensing layer. Since the vertical measurement tool and the LED used a radiation of the same wavelength, the laser light is reflected, allowing for vertical displacement measurements; additionally, LED light is optimally reflected and confined to the sensing membrane, linearizing the characteristic and resulting in a broader measurement range. These results suggest that the use of a biocompatible paint in future studies might improve the behaviour of the sensor.

## 5. Discussion and Future Work

In this work, a new sensing mechanism for stretches is proposed, by considering an LED, a photodiode and the processing electronics. Compared with the state of the art, the required fabrication process is simpler and does not need any advanced instrumentation [5], and the proposed measurement technique is more affordable than [30] , considering its cheaper measurement setup and easier post-processing of data. Furthermore, from [23], mechanical improvements were made, since the stretches are applied through a soft membrane deformation in this proposal, and not through the mechanical displacement of two parts, reducing the breaking risk as fewer mechanical stresses are present. Moreover, the optical fibers are now isolated from the culture medium, reducing unwanted optical effects at the interfaces. The sensing technique (i.e., using the PDMS as an optical waveguide) was tested to check its suitability for a proposed application. The findings indicate that, with the current setup, stretches of up to 1.5% were experimentally measured, but simulations point towards the possibility of expanding the technique to stretches of up to 10–30%. These considerations open the possibility of many research lines and future work, where better light confinement should be sought in the sensing layer. For instance, SU-8 is a biocompatible [36] photoresist that is widely employed in the MEMS field and is highly reflective of the infrared wavelengths [37]. The effects of these material coatings on the sensor characteristic could be investigated to improve the optical coupling and increase the sensing range.

## 6. Conclusions

In this article, an innovative stretch sensing mechanism has been proposed, where a thin PDMS membrane is used as an optical waveguide and tested for a specific application. The results obtained from this proof-of-concept have demonstrated that, with a simple and cost-effective fabrication process, the detecting mechanism is capable of measuring stretches of up to 1.5% at the device-sensing layer at low pressures. Simulations point towards the possibility of expanding the range to up to 10–30% stretch with higher pressures. Painting the top layer of the membrane improves the linearity and the sensing range of the membrane, opening future lines of investigation regarding this aspect by enhancing the fabrication method with the use of more precise instrumentation and coatings.

## Data Availability

All the data generated/analyzed in this study are included in this article itself.
